# Case Series of Steroid-Resistant Immune Checkpoint Inhibitor Associated Myocarditis: A Comparative Analysis of Corticosteroid and Tofacitinib Treatment

**DOI:** 10.3389/fphar.2021.770631

**Published:** 2021-12-06

**Authors:** Cong Wang, Jinyi Lin, Yan Wang, David H. Hsi, Jiahui Chen, Tianshu Liu, Yuhong Zhou, Zhenggang Ren, Zhaochong Zeng, Leilei Cheng, Junbo Ge

**Affiliations:** ^1^ Department of Cardiology, Zhongshan Hospital, Shanghai Institute of Cardiovascular Diseases, Fudan University, Shanghai, China; ^2^ Department of Oncology, Zhongshan Hospital, Fudan University, Shanghai, China; ^3^ Department of Cardiology, Stamford Hospital, Stamford, CT, United States; ^4^ Department of Liver Medicine, Zhongshan Hospital, Fudan University, Shanghai, China; ^5^ Department of Radiotherapy, Zhongshan Hospital, Fudan University, Shanghai, China; ^6^ Department of Echocardiography, Zhongshan Hospital, Shanghai Institute of Cardiovascular Diseases, Shanghai Institute of Medical Imaging, Fudan University, Shanghai, China

**Keywords:** immune checkpoint inhibitor, myocarditis, corticosteroid, tofacitinib, cardiotoxicity

## Abstract

**Background:** Immune checkpoint inhibitor (ICI)–associated myocarditis is an uncommon and potentially fatal immune-related adverse event (irAE). Although corticosteroids are recommended as the first-line treatment by current guidelines, patients still have variable responses to it, and the guidelines vary significantly in terms of treatment strategies.

**Objectives:** In this study, we performed a retrospective analysis of ICI-associated myocarditis in our hospital to propose a new comparative analysis to aid individualized treatment.

**Methods:** We reviewed detailed records of 24 patients with confirmed ICI-associated myocarditis in our hospital from July 1, 2019, to April 1, 2021. Although all the cases in our study received recommended initial corticosteroid treatment according to the guidelines, different responses to corticosteroid were observed during the process of subsequent corticosteroid tapering. Basing on troponin cardiac troponin T rebound during corticosteroid tapering, we propose a new classification analysis of ICI-associated myocarditis that included two subgroups: corticosteroid-sensitive (n = 8) and corticosteroid-resistant group (n = 16).

**Results:** Compared with corticosteroid-sensitive patients, larger doses of corticosteroid, longer period of treatment, and higher mortality rate were found in corticosteroid-resistant patients. Corticosteroid-resistant patients were characterized by more prominent ptosis, muscle weakness, elevated cardiac biomarkers, creatine kinase, and hepatic enzymes levels than that in the corticosteroid-sensitive patients. Tofacitinib (5 mg twice a day) was used in 11 corticosteroid-resistant patients, with seven patients recovered from ICI-associated myocarditis, showing a promising therapeutic effect.

**Conclusion:** Our group analysis of corticosteroid responsiveness in patients with ICI-associated myocarditis may help clinicians to apply individualized treatment in this high-risk cohort. In addition, tofacitinib could provide clinical benefits when used early in the corticosteroid-resistant patients and may provide a new option for the treatment of ICI-associated myocarditis.

## Introduction

Immune checkpoint inhibitors (ICIs) including monoclonal antibodies (mAbs) against cytotoxic T-lymphocyte–associated antigen 4 (CTLA-4), programmed cell death protein 1 (PD-1), and programmed cell death ligand 1 (PD-L1) have significantly improved cancer treatment and achieved unprecedented efficacy in some types of cancer (Sasidharan Nair and Elkord, 2018; Togashi et al., 2019). Since the advent of ipilimumab (CTLA-4 mAbs), the first ICI approved by the US Food and Drug Administration (FDA) in 2011, ICIs are routinely used in clinical treatments. There are more than 1,200 ICI-associated registered trials worldwide (Topalian, 2017; Ribas and Wolchok, 2018). With the growing indications of ICIs and their fundamental role in cancer therapies, more ICI-associated side effects have drawn attention of clinicians.

The mechanisms of ICIs are based on promoting T-cell–mediated antitumor activity by targeting the intrinsic immune “brakes” (immune checkpoints). Blockade of these inhibitory pathways by targeting PD-1, PD-L1, and CTLA-4 releases the brakes of tumor-reactive T cells antitumor activity and leads to the remarkable clinical benefit (Kallies et al., 2020; Waliany et al., 2021). Unfortunately, autoreactive T cells may also be inappropriately activated by ICIs, leading to a broad spectrum of adverse events termed immune-related adverse events (irAEs). Because of their widespread effects on the immune system, irAEs can influence almost every organ, including the colon, lungs, liver, skin, thyroid, and heart (Michot et al., 2016; Postow et al., 2018). ICI-related cardiotoxicity is uncommon but characterized by a high mortality rate (Wang et al., 2018). Previous studies have reported some types of cardiovascular irAEs, including myocarditis, pericardial disease, arrhythmia, acute coronary syndrome, and vasculitis (Khunger et al., 2020; Baik et al., 2021). Since the FDA approval of ipilimumab in 2011, six more ICIs have been approved for cancer therapy including nivolumab, pembrolizumab, cemiplimab atezolizumab, avelumab, and durvalumab (Vaddepally et al., 2020). Although the incidence of ICI-associated myocarditis is merely approximately 1%, the mortality rate of myocarditis is up to 50% despite intensive treatment (Al-Kindi and Oliveira, 2018; Anquetil et al., 2018; Palaskas et al., 2020). Both cardiologists and oncologists need to be familiar with ICI-associated myocarditis and treatment options.

Because of the large variability of clinical symptoms, there is no consensus on standard regimens of ICI-associated myocarditis currently. The treatment of ICI-associated myocarditis has largely been based on the therapy of viral myocarditis (Palaskas et al., 2020). Corticosteroids are usually the first-line treatment. While the early identification of corticosteroid effect is crucial for further therapy, there have been case reports of successfully treated ICI-associated myocarditis with intravenous immunoglobulin, mycophenolate, infliximab, plasmapheresis, and alemtuzumab (Arangalage et al., 2017; Norwood et al., 2017; Frigeri et al., 2018; Esfahani et al., 2019). However, the effectiveness of these agents is still unclear because of inadequate response to corticosteroids in many patients.

Currently, the severity of ICI-associated cardiotoxicity can be divided into four grades: grade 1 is the mildest (asymptomatic with laboratory abnormalities), and grade 4 is the most severe (moderate to severe cardiac impairment and life-threatening conditions) (Brahmer et al., 2018). However, this classification system may not clearly specify response to corticosteroid therapy. Mortality rate remained high in the commonly used corticosteroid therapy (Zhang et al., 2020). In this study, we proposed a new classification for ICI-associated myocarditis including two subgroups: corticosteroid-sensitive and corticosteroid-resistant group, according to the clinical presentations and outcomes, to guide the individualized treatment options. In this study, tofacitinib treatment was used in 11 cases of ICI-associated myocarditis who responded poorly to corticosteroid and immunosuppressive regiments. Tofacitinib demonstrated promising treatment effects for ICI-associated myocarditis.

## Methods

### Patient Selection

This retrospective study was conducted in Zhongshan Hospital of Fudan University. We identified patients who received ICI treatment between July 2019 and April 2021. The inclusion criteria are as follows: (1) definitive diagnosis of myocarditis such as abnormal cardiac magnetic resonance imaging (CMR), clinical syndrome of myocarditis, and positive biomarkers; (2) complete medical history and follow-up; and (3) endpoint events—cardiac recovery or death. Data extracted from the medical records included demographics, clinical presentation, medical treatment for myocarditis, laboratory data, discontinuation or withholding of ICIs, and patients’ clinical outcomes. The study was approved by the Zhongshan Hospital Institutional Review Board.

### Treatment and Evaluation

Once the diagnosis of myocarditis was identified, all patients in our study received recommended corticosteroid treatment according to the current guidelines (Brahmer et al., 2018; Haanen et al., 2018). To be specific, in mild cases (grade 1–2), 1 to 2 mg/kg methylprednisolone was given intravenously. The tapering period lasted for 4 to 6 weeks. For moderate to severe decompensation cases (grade 3–4), pulse therapy was administered by giving methylprednisolone 500 to 1,000 mg/d plus either antithymocyte globulin or other immune suppressants such as infliximab. For subsequent corticosteroid tapering, methylprednisolone was used 1 to 2 mg/kg/day for 3 days and then reduced by 10 to 20 mg every 3 to 5 days to 40 mg before changing into oral prednisolone finally. For pulse therapy, methylprednisolone was used in order of decreasing doses as follows: 500 mg for 3 days, 240 mg for 3 days, 120 mg for 3 days, 80 mg for 3 days, 60 mg for 3 days, and 40 mg for 3 days and then changed into oral prednisolone.

Janus kinase (JAK) pathway inhibitor tofacitinib was used in patients whose cardiac troponin T (cTnT) levels started to increase during corticosteroid tapering. We monitored patients closely by measuring troponin, creatine kinase, and renal and liver function every day or every other day. After reaching the plateau, these indicators were checked every 2 weeks together with electrocardiogram and echocardiography. Standard myocardial protection and antimyocardial remodeling medications such as coenzyme Q10, β-blockers, angiotensin-converting enzyme inhibitors, or angiotensin II receptor blockers were used in all patients with ICI-associated myocarditis. We tried to titrate all medications to the target dosages.

### Statistical Analysis

Kaplan–Meier method was utilized to estimate the overall survival and differences in survival curves between the corticosteroid-sensitive and corticosteroid-resistant groups. Differences between groups were tested by Student *t* test. All statistical tests were two-sided with an α level of 0.05. Statistical analysis was performed by using SPSS version 19.

## Results

### Baseline Characteristics

**FIGURE 1:**
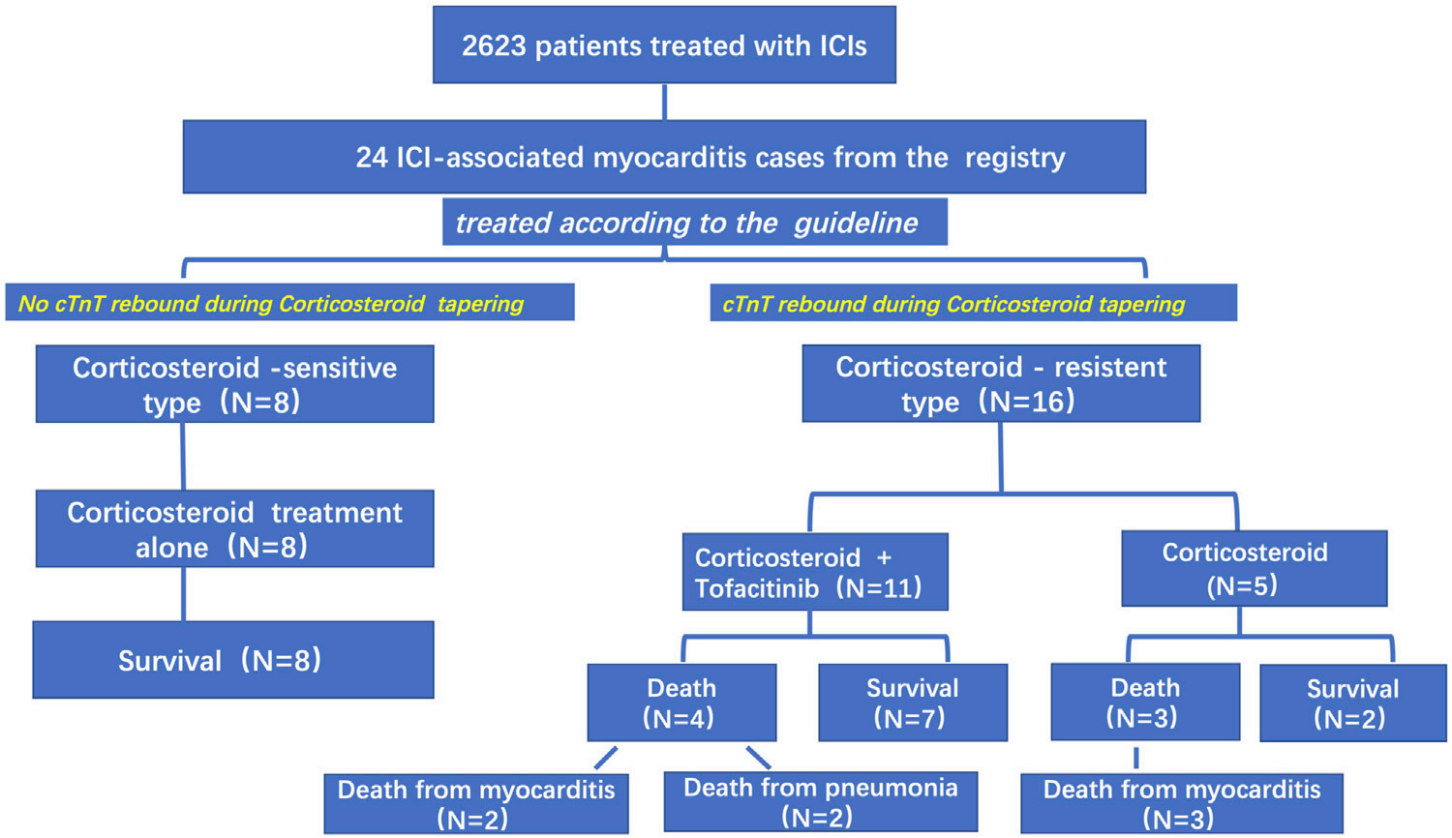
Treatment for ICI-associated myocarditis. Based on the response to corticosteroids (troponin cTnT levels rebounding or not during corticosteroid tapering), we divided patients with ICI-associated myocarditis into corticosteroid-sensitive and corticosteroid-resistant groups.

**FIGURE 2:**
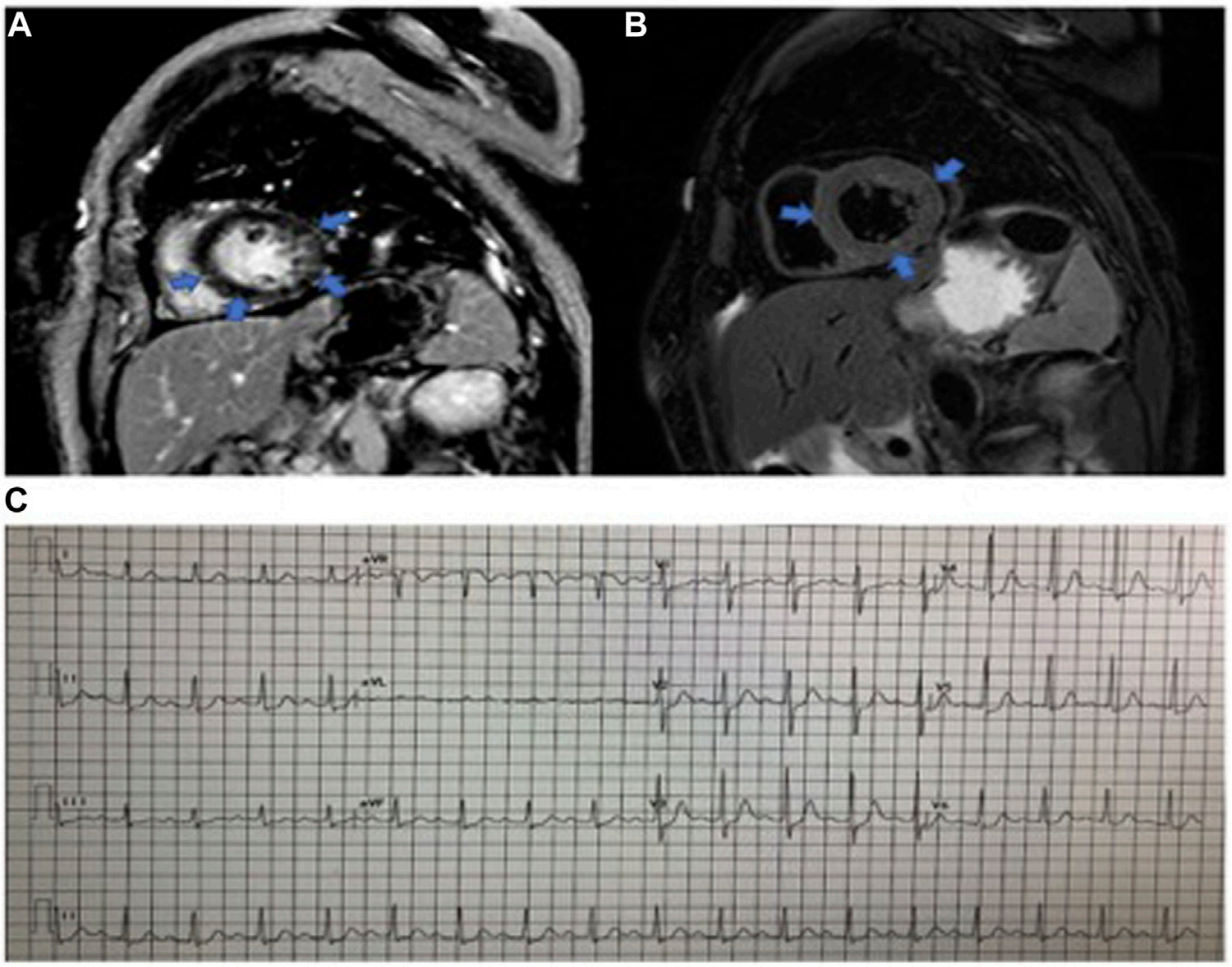
Cardiac magnetic resonance imaging of myocarditis. **(A)** LGE image of fibrosis. **(B)**. Precontrast T2-weighted image of the same slice location as the image in the same patient. **(C)**. Eelectrocardiogram abnormalities: the prolongation of the PR interval, sinus tachycardia, and ST changes.

From July 1, 2019, to April 1, 2021, 2,623 patients in our center were treated with PD-1 or PD-L1 mAbs (Figure 1). Based on the inclusion criteria, 24 ICI-associated myocarditis patients (16 males and 8 females) were included in this study. ICI agents applied included pembrolizumab, toripalimab, sintilimab, cadonilimab, durvalumab and camrelizumab. Among them, four patients were treated with ICIs only while 20 patients were treated with ICIs plus chemotherapy or radiation therapy. Demographic and baseline clinical characteristics of these patients are presented in Table 1. The most common cancer diagnosis was gastric, hepatic, and colorectal cancer. All patients were in the stage 3 to 4 of cancer. The most common initial clinical presentation in our cohort was malaise, followed by chest congestion, muscular soreness, and ptosis. Electrocardiographic abnormalities were detected in 18 patients (75%) including atrial or ventricular premature beats, sinus tachycardia, sinus bradycardia, ST-T changes, and atrial fibrillation. No patients had high-grade atrioventricular block or malignant arrhythmia. Echocardiographic abnormalities, such as left ventricular hypertrophy, left atrial enlargement, and valvular regurgitation, were detected in 11 patients. The most common CMR finding was late gadolinium enhancement (LGE), whereas functional abnormalities were detected in three patients (Figure 2).

**TABLE 1:** Baseline characteristics in patients with ICI-associated myocarditis.

Case	Age	Gender	Malignancy	Onset symptoms	Cardiovascular complications	Associated irAEs	ECG	TTE	CMR
1	66	Male	Gastric carcinoma	Asymptomatic	Atrial fibrillation	Creatine kinase elevation	Atrial fibrillation	Atrial enlargement, mild mitral regurgitation	Local edema and LGE
2	72	Male	Gastric carcinoma	Asymptomatic		Creatine kinase elevation	RBBB	No significant abnormality	Local edema and LGE
3	70	Female	Hepatic carcinoma	Asymptomatic		Creatine kinase elevation	Sinus rhythm	No significant abnormality	Local edema
4	67	Male	Hepatic carcinoma	Asymptomatic		Creatine kinase elevation	Sinus rhythm	Ventricular septal hypertrophy	Ventricular septal hypertrophy
5	66	Female	Colorectal carcinoma	Asymptomatic	Coronary artery fistula		Sinus rhythm	No significant abnormality	
6	72	Male	Lung carcinoma	Asymptomatic			Atrial premature beats	Aortic regurgitation	Local edema and LGE
7	63	Male	Gastric carcinoma	Asymptomatic			Sinus bradycardia	BAV	Local edema and LGE
8	38	Female	Leiomyosarcoma	Asymptomatic			Sinus tachycardia	No significant abnormality	Local edema and LGE
9	71	Male	Gastric carcinoma	Ptosis, muscle soreness, malaise		Creatine kinase elevation	Atrial premature beats	Dilated aorta	Local edema and LGE
10	68	Female	Hodgkin lymphoma, breast carcinoma	Chest congestion	Hypertension	Creatine kinase elevation	Atrial premature beats	No significant abnormality	Local LGE
11	57	Female	Cervical carcinoma	Chest congestion		Thyroiditis	ST changes	LVEF <50%	Wide LGE
12	65	Male	Head-neck carcinoma	Malaise		Creatine kinase elevation	T changes	Atrial enlargement, dilated aorta	Local edema and LGE
13	54	Male	Colorectal carcinoma	Malaise		Hepatitis	ST changes	Atrial enlargement	Local edema and LGE
14	63	Female	Gastric carcinoma	Ptosis, malaise		Creatine kinase elevation	T changes	Mild mitral regurgitation	No significant inflammation
15	71	Female	Esophageal carcinoma	Ptosis, muscle soreness		Creatine kinase elevation	First-degree AV block	No significant abnormality	
16	63	Female	Malignant melanoma	Ptosis, malaise		Creatine kinase elevation	Sinus rhythm	No significant abnormality	No significant inflammation
17	58	Male	Gastric carcinoma	Ptosis, malaise		Creatine kinase elevation	RBBB	LVEF <50%	
18	60	Male	Esophageal carcinoma	Malaise		Creatine kinase elevation	Sinus rhythm	Minimal pericardial effusion	Local edema
19	59	Male	Colorectal carcinoma	Ptosis, malaise	Hypertension	Creatine kinase elevation	Sinus rhythm	Atrial enlargement	Local edema and LGE
20	66	Male	Hepatic carcinoma	malaise		Creatine kinase elevation	ST changes	Dilated aorta; mild aortic regurgitation	Local edema and LGE
21	65	Male	Lung carcinoma	Ptosis, malaise			RBBB; ST changes; ventricular premature beat	Minimal pericardial effusion	Local edema and LGE
22	77	Male	Hepatic carcinoma	Ptosis, malaise	Hypertension	Creatine kinase elevation	Sinus tachycardia; first-degree AV block	Atrial enlargement	
23	53	Male	Hepatic carcinoma	Chest congestion, malaise		Creatine kinase elevation	Sinus tachycardia	Atrial enlargement	Local edema and LGE
24	41	Male	Hepatic carcinoma	malaise		Creatine kinase elevation	Sinus tachycardia; ST changes	LVEF <50%; moderate mitral regurgitation	Wide LGE

BAV, bicuspid aortic valve; ECG, electrocardiogram; LVEF, left ventricular ejection fraction; RBBB, right bundle-branch block; TTE, transthoracic echocardiogram.

### The Comparative Analysis Based on Corticosteroid Responsiveness

Although all the cases in this study received recommended initial corticosteroid treatment according to the guidelines (Brahmer et al., 2018; Haanen et al., 2018), different responses to corticosteroid were observed in subsequent tapering. Troponin cTnT levels of eight patients gradually decreased accompanied by decrement of corticosteroid. However, the levels of troponin cTnT rebounded in 16 patients during corticosteroid tapering despite symptomatic remission after the initial treatment.

Based on troponin cTnT rebound during corticosteroid tapering, we classified patients with ICI-associated myocarditis into two subgroups: (1) corticosteroid-resistant ICI-associated myocarditis: troponin cTnT rebound during corticosteroid tapering; and (2) corticosteroid-sensitive ICI-associated myocarditis: no troponin cTnT rebound during corticosteroid tapering.

Eight corticosteroid-sensitive patients responded favorably to the treatment evidenced by gradually troponin cTnT decreasing, smooth corticosteroid tapering, and shorter treatment time. In 16 corticosteroid-resistant patients, troponin cTnT increased again and required high-dose corticosteroid regimen with rapid tapering. Troponin cTnT rebound often occurred during second to third corticosteroid tapering. In this study, the initial methylprednisolone doses of corticosteroid-sensitive patients were approximately 1 to 2 mg/kg per day, and total intravenous dose was less than 1,500 mg. For corticosteroid-resistant patients, initial methylprednisolone dose was usually 500 mg, and total intravenous dose was much higher than that in the corticosteroid sensitive-group (Table 2).

**TABLE 2:** Treatment of 24 patients with ICI-associated myocarditis.

Case	ICIs	Time to onset (days)	Combined anticancer drug	Combined therapy	Initial cTnT (ng/mL) (<0.003 ng/mL)	Time to corticosteroid treatment (days)	Initial corticosteroid dose	Total intravenous corticosteroid dose (mg)	Time to cTNT recovery (days)	Time to corticosteroid finished (days)	Outcome
1	Camrelizumab (anti–PD-1)	14	Oxaliplatin + 5-fluorouracil		0.094	5	Methylprednisolone 160 mg	1,280	51	72	Improved
2	Camrelizumab (anti–PD-1)	14			0.1	19	Methylprednisolone 120 mg	660	100	86	Improved
3	Sintilimab (anti–PD-1)	21			0.229	9	Methylprednisolone 80 mg	600	97	68	Improved
4	Pembrolizumab (anti–PD-1)	21			0.182	1	Methylprednisolone 50 mg	950	34	60	Improved
5	Pembrolizumab (anti–PD-1)	42	Regorafenib		0.033	5	Methylprednisolone 120 mg	930	27	61	Improved
6	Sintilimab (anti–PD-1	21	Paclitaxel + cisplatin		0.086	2	Methylprednisolone 40 mg	200	5	51	Improved
7	Cadonilimab (anti–PD-1/CTLA-4)	56	Oxaliplatin + capecitabine		0.05	2	Methylprednisolone 120 mg	540	3	65	Improved
8	Sintilimab (anti–PD-1)	63	Eribulin		0.136	7	Methylprednisolone 120 mg	540	5	67	Improved
9	Camrelizumab (anti–PD-1)	28	Oxaliplatin + capecitabine	Immunoglobulin + tofacitinib	0.507	1	Methylprednisolone 500 mg	3,090	104	107	Improved
10	Sintilimab (anti–PD-1)	21		Immunoglobulin + tofacitinib + plasmapheresis	0.508	8	Methylprednisolone 500 mg	2,880			Death from pneumonia
11	Pembrolizumab (anti–PD-1)	147	Bevacizumab + paclitaxel + cisplatin		0.328	6	Methylprednisolone 240 mg	1,660			Death from myositis progression
12	Toripalimab (anti–PD-1)	14	Gemcitabine	Infliximab 500 mg	0.072	1	Methylprednisolone 500 mg	4,000			Death from myositis progression
13	Camrelizumab (anti–PD-1)	28	Fruquintinib	Immunoglobulin + tofacitinib	0.048	2	Methylprednisolone 500 mg	3,120			Death from myositis progression
14	Camrelizumab (anti–PD-1)	42		Immunoglobulin + tofacitinib	0.348	6	Methylprednisolone 500 mg	3,330	118	121	Improved
15	Camrelizumab (anti–PD-1)	14	Paclitaxel		0.332	6	Methylprednisolone 80 mg	320			Death from myositis progression
16	Toripalimab (anti–PD-1	28		Immunoglobulin + tofacitinib	1.44	1	Methylprednisolone 40 mg	240	93	87	Improved
17	Camrelizumab (anti–PD-1)	14	Apatinib	Immunoglobulin + tofacitinib	2.09	10	Methylprednisolone 500 mg	3,500			Death from pneumonia
18	Cadonilimab (anti–PD-1/CTLA-4)	42		Immunoglobulin + tofacitinib	0.113	16	Methylprednisolone 500 mg	3,000	64	70	Improved
19	Pembrolizumab (anti–PD-1)	21	Fruquintinib	Immunoglobulin + tofacitinib	0.165	1	Methylprednisolone 240 mg	1,500	106	111	Improved
20	Pembrolizumab (anti–PD-1)	21	Lenvatinib	Tofacitinib	0.06	2	Methylprednisolone 80 mg	1,500	50	69	Improved
21	Sintilimab (anti–PD-1)	42	Cisplatin + docetaxel	Immunoglobulin	0.731	11	Methylprednisolone 200 mg	2,320	70	91	Improved
22	Toripalimab (anti–PD-1)	14	Bevacizumab	Immunoglobulin + tofacitinib	2.86	1	Methylprednisolone 500 mg	3,360			Death from myositis progression
23	Sintilimab (anti–PD-1)	63	Lenvatinib	Tofacitinib	0.403	21	Methylprednisolone 60 mg	540	55	70	Improved
24	Durvalumab (anti–PD-L1)	42	Bevacizumab	Immunoglobulin	0.122	2	Methylprednisolone 500 mg	3,900	62	98	Improved

In addition, infliximab (500 mg in one patient) and tofacitinib (5 mg twice a day in 11 patients) were used in corticosteroid-resistant patients. Interestingly, tofacitinib achieved a satisfactory therapeutic effect. Seven patients received tofacitinib treatment recovered from ICI-associated myocarditis, whereas two patients died of severe pneumonia, and the other two patients died of myocarditis progression.

### Differences Between Two Groups

There is no significant difference of age and gender between corticosteroid-sensitive and corticosteroid-resistant group. According to the American Society of Clinical Oncology guidelines (Brahmer et al., 2018), myocarditis severity in corticosteroid-sensitive group was between grade 1 and grade 2. For corticosteroid-resistant group, grades of severity were between grade 2 and grade 3. Patients in the corticosteroid-resistant group had frequent symptoms of chest congestion, muscular soreness, and ptosis. Biomarkers troponin cTnT and creatine phosphokinase (CPK) were increased more significantly in the corticosteroid-resistant group than in the corticosteroid-sensitive group (Table 3). Our data revealed that corticosteroid-resistant patients have more complications such as myositis and autoimmune hepatitis. Therefore, the levels of aspartate aminotransferase (AST), alanine aminotransferase (ALT), and C-reactive protein were increased more in corticosteroid-resistant patients than in corticosteroid-sensitive group patients.

**TABLE 3:** Comparison of corticosteroid-sensitive and corticosteroid-resistant ICI-associated myocarditis patients.

Characteristics	Corticosteroid-sensitive (n = 8)	Corticosteroid-resistant (n = 16)	*p*
Age (years)	64.3 ± 3.9	61.9 ± 2.1	NS
Gender (M/F)	5/3	11/5	NS
ST2 (ng/mL)	69.1 ± 14.2	116.9 ± 31.1	NS
BNP (pg/mL)	244.0 ± 94.8	1,498.4 ± 531.3	NS
cTNT (ng/mL)	0.11 ± 0.02	0.63 ± 0.2	<0.05
CK (U/L)	384.6 ± 139.2	2,326.8 ± 654.4	<0.05
CKMB (U/L)	33.1 ± 7.5	125.9 ± 26.8	<0.05
CKMM (U/L)	351.4 ± 135.5	2,361.1 ± 665.0	<0.05
ALT (U/L)	33.3 ± 9.2	144.6 ± 28.3	<0.05
AST (U/L)	48.0 ± 11.9	182.8 ± 46.2	<0.05
CRP (mg/L)	4.4 ± 1.8	64.4 ± 25.2	<0.05

BNP, brain natriuretic peptide; CK, creatine kinase; NS, no significance; ST2, suppression of tumorigenicity 2.

### Survival

**FIGURE 3:**
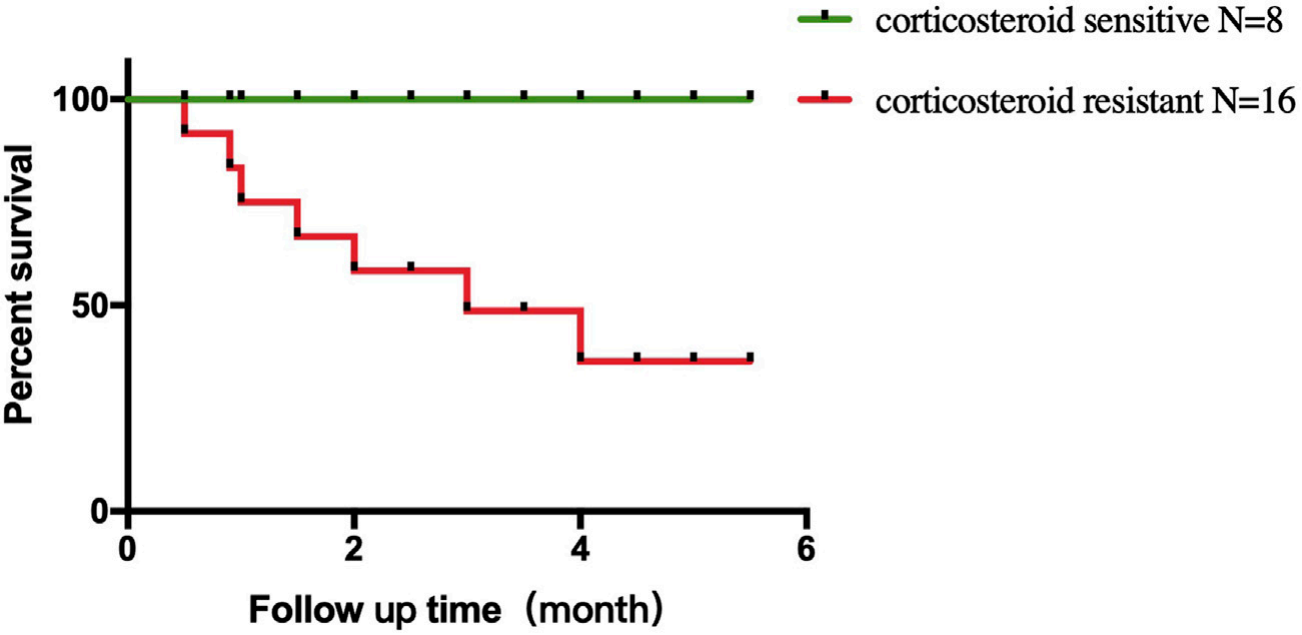
Comparison of survival curves. Higher mortality in corticosteroid-resistant patients compared with corticosteroid-sensitive patients.

All eight corticosteroid-sensitive patients recovered from ICI-associated myocarditis. In the corticosteroid-resistant group, nine patients recovered from ICI-associated myocarditis (recovery rate = 56.3%), whereas the other seven patients died. The reason of death could be listed as follows: two patients died of septic shock due to severe pneumonia, and five patients died of cardiogenic shock due to the progression of myocarditis. Comparison of survival curves using log-rank test demonstrated a higher mortality in corticosteroid-resistant patients than that in the corticosteroid-sensitive patients (*p* = 0.01) (Figure 3).

## Discussion

In this study, we reported 24 ICI-associated myocarditis patients and analyzed some important clinical parameters. Based on the response to corticosteroids (troponin cTnT levels rebounding or not during corticosteroid tapering), we divided ICI-associated myocarditis patients into corticosteroid-sensitive and corticosteroid-resistant group. Corticosteroid-resistant patients could benefit from tofacitinib treatment. This illustrated the heterogeneity in etiology and pathophysiology in these patients. Response to corticosteroid treatment may predict the prognosis, guide the corticosteroid doses, and initiate additional immune suppression therapy (Table 4).

**TABLE 4:** ICI-associated myocarditis: corticosteroid-sensitive and corticosteroid-resistant sub-type.

	Corticosteroid-sensitive	Corticosteroid-resistant
Characteristic	cTnT decrease during reduction of corticosteroid dose	cTnT rebound during reduction of corticosteroid dose
Symptoms	Asymptomatic or mild symptoms	Ptosis, muscle soreness, malaise
Laboratory tests	Mild to moderate increase of myocardial injury biomarker, hepatic enzymes, CPK, inflammation biomarker	Significant increase of myocardial injury biomarker, hepatic enzymes, CPK, inflammation biomarker
CMR	No significant abnormality	Edema and LGE
Treatment initial corticosteroid dose	Methylprednisolone 1–2 mg/kg per day	Methylprednisolone 500 mg × 3 days
Additional therapy		Tofacitinib 5 mg bid

Previously, ICI-associated myocarditis had been reported with an incidence of 0.03% to 1.14% (Mahmood et al., 2018; Salem et al., 2018). Recent studies have indicated an increase in its incidence, which may be related to the expanding use of ICIs (Al-Kindi and Oliveira, 2018; Salem et al., 2018). Up until now, the precise mechanisms of ICI-associated myocarditis remain unclear. Suggested possibilities include shared antigens between the tumor and myocardium, T-cell receptor targeting homologous muscle antigen as the tumor antigen, or certain T-cell receptors targeting dissimilar antigens (Lv et al., 2011; Johnson et al., 2016; Khunger et al., 2020). Thus, T cells targeting the shared epitopes between tumor and myocardium may exist, and the ICIs can augment the T-cell effector function, resulting in the development of autoimmune myocarditis (Müller et al., 2018; Tocchetti et al., 2018). The degree and spectrum of inflammation may account for the variable clinical presentations and treatment effects.

**FIGURE 4:**
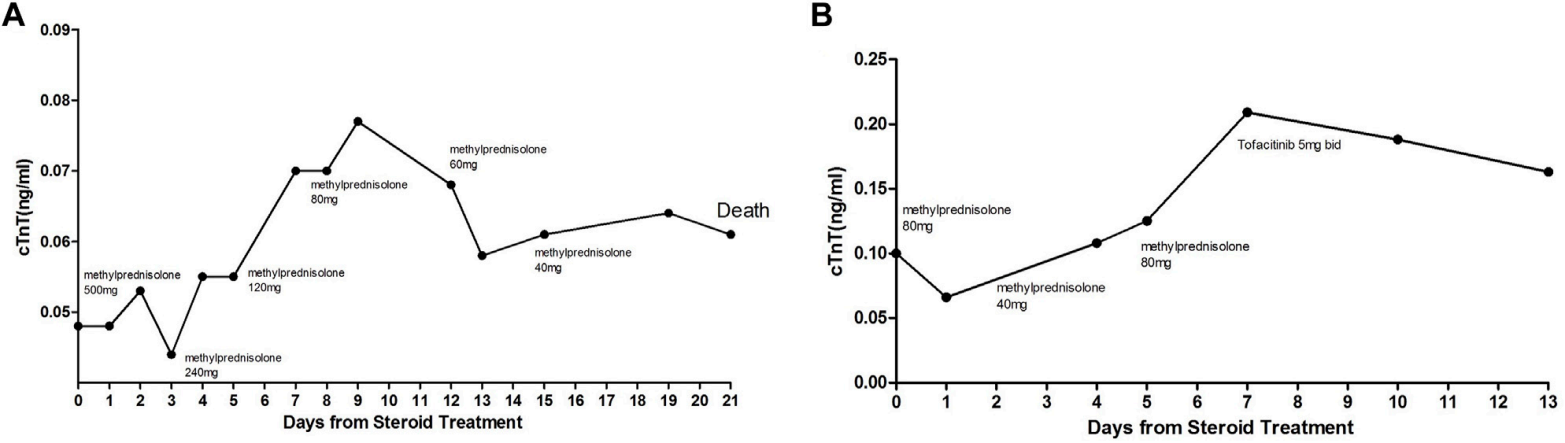
Two cases from the corticosteroid-resistant group. Initial cTnT levels were less than 0.1 ng/mL; however, cTnT levels rebounded significantly during corticosteroid tapering.

The treatment of ICI-associated myocarditis has largely been based on the corticosteroids. According to the recommendations of the Society for Immunotherapy of Cancer, four grades of cardiovascular irAEs have been defined and used to guide initial corticosteroid doses (Puzanov et al., 2017). It is still difficult to predict the prognosis even if high-dose corticosteroid regimen was used. cTnT is a cardiac-specific biomarker of ongoing myocardial inflammation in myocarditis. Therefore, monitoring the changes of troponin T levels provides a reliable way to assess clinical situation in ICI-associated myocarditis cases. We would like to point out that our classification is based on the change of cTnT levels rather than the initial cTnT levels. As shown in Figure 4, two cases from the corticosteroid-resistant group had initial cTnT levels of less than 0.1 ng/mL, whereas the cTnT levels rebounded remarkably during corticosteroid tapering. Therefore, troponin cTnT rebound during corticosteroid tapering probably reflected ongoing myocardial inflammation and necrosis.

Once ICI-associated myocarditis was diagnosed, we used intravenous methylprednisolone 1 to 2 mg/kg daily before transition to oral prednisone 1 to 2 mg/kg per day followed by tapering doses over several weeks for corticosteroid-sensitive patients. For corticosteroid-resistant patients, we recommend intravenous methylprednisolone 500 mg daily before transition to oral prednisone 1 to 2 mg/kg daily. Other immunosuppressants were used in different centers including mycophenolate mofetil, antithymocyte globulin, and intravenous immunoglobulin (Arangalage et al., 2017; Norwood et al., 2017; Tay et al., 2017). One additional option for immunosuppression is the chimeric immunoglobulin G mAb to tumor necrosis factor α (TNF-α), named infliximab. By binding to TNF-α, a major proinflammatory cytokine, infliximab, leads to the downregulation of other cytokines and induces the apoptosis of TNF-producing cells, including T lymphocytes (Frigeri et al., 2018; Khunger et al., 2020). One of our corticosteroid-sensitive patients and five corticosteroid-resistant patients received combined ICI agents and tyrosine kinase inhibitor (TKI) treatment. There is a strong impetus for combining TKI and ICI, given their complementary response profile and synergy in generating antitumor immunity. It has been demonstrated that ICI in conjunction with TKI enhanced efficacy in multiple tumor types (Ascierto et al., 2019; Rini et al., 2019). As both agents could cause cardiotoxicities, it is therefore possible that a higher rate of cardiotoxicity might be observed with combination regimens. Future clinical trials combining ICI and TKI should prospectively assess biomarkers of cardiotoxicity for better clinical understanding and comprehensive assessment.

Compared with traditional treatment modalities, we studied tofacitinib with interesting results. It is a JAK inhibitor, which blocks the production of proinflammatory cytokines through the suppression of JAK–signal transducer and activator of transcription (STAT) signal pathway (Angelini et al., 2020; Hosseini et al., 2020). It has been already used for rheumatoid arthritis, psoriatic arthritis, and ulcerative colitis (Moura and Fonseca, 2020; Chimenti et al., 2021). Increased expression of phosphorylated proteins of JAK-STAT pathway was observed in rat autoimmune myocarditis model; while being treated with JAK inhibitor, the cardiac function and myocardial inflammation were alleviated (Liu et al., 2016). A previous study proved that JAK/STAT signaling pathway played a vital role in the tumorigenesis and could promote tumor evasion by conferring high PD-L1 expression on tumor cells (Luo et al., 2018; Song et al., 2018). Therefore, targeting JAK/STAT pathway might also have a synergistic antitumor effect of ICI therapy. Tofacitinib was the first small molecule JAK inhibitor; it reversibly and competitively binds to the ATP binding site of the kinase domain of JAK (Flanagan et al., 2010). Compared with specific inflammatory pathway mAb, tofacitinib is a pan-JAK inhibitor, effecting JAK1, JAK2, JAK3, TYK2, IL-6, and type I interferons (Fernández-Clotet et al., 2018). Given the potential risks of inflammatory cytokine storm in corticosteroid-resistant patients, it is reasonable to observe the favorable results after tofacitinib treatment. Therefore, our findings may provide a new option for clinical treatment of refractory myocarditis confirmed by other investigators.

## Limitation

Significant differences of clinical symptoms and laboratory and imaging tests could be observed between the two groups. Because of the limited cases, we were unable to construct a scoring system to predict the efficacy of corticosteroid therapy. Our initial experience was from a small and retrospective study without comparison to other possible treatment regimens. To confirm the validity of myocarditis treatment strategies in corticosteroid-sensitive and corticosteroid-resistant groups, a prospective and randomized clinical trial enrolling large patient samples will be of critical importance in the future.

## Conclusion

Corticosteroid responsiveness in patients with ICI-associated myocarditis may guide clinicians to provide targeted treatment in this high-risk cohort. Based on our retrospective study, tofacitinib could offer additional clinical benefits when being used early in the corticosteroid-resistant patients and provide a new option for the treatment of ICI-associated myocarditis.

Clinical Perspectives: This new group analysis for ICI-associated myocarditis may guide individualized therapies in this high-risk population. Tofacitinib treatment may have impactful clinical benefits when being used early in corticosteroid-resistant patients.

## Data Availability

The raw data supporting the conclusion of this article will be made available by the authors, without undue reservation.

## Abbreviations

ICIs, immune checkpoint inhibitors; CTLA-4, cytotoxic T-lymphocyte–associated antigen 4; PD-1, programmed cell death protein 1; PD-L1, programmed cell death ligand 1; irAEs, immune-related adverse events; CMR, cardiac magnetic resonance imaging; JAKs, Janus kinases; LGE, late gadolinium enhancement; CPK, creatine phosphokinase; BNP, brain natriuretic peptide; AST, aspartate aminotransferase; ALT, alanine aminotransferase; TNF-α, tumor necrosis factor α; TKI, tyrosine kinase inhibitors; STAT, signal transducer and activator of transcription
